# A Durable Response With the Combination of Nivolumab and Cabozantinib in a Patient With Metastatic Paraganglioma: A Case Report and Review of the Current Literature

**DOI:** 10.3389/fendo.2020.594264

**Published:** 2020-11-27

**Authors:** Minas P. Economides, Amishi Y. Shah, Camilo Jimenez, Mouhammed A. Habra, Monica Desai, Matthew T. Campbell

**Affiliations:** ^1^ Department of Internal Medicine, The University of Texas School of Health Sciences at Houston, Houston, TX, United States; ^2^ Department of Genitourinary Medical Oncology, The University of Texas MD Anderson Cancer Center, Houston, TX, United States; ^3^ Department of Endocrine Neoplasia and Hormonal Disorders, The University of Texas MD Anderson Cancer Center, Houston, TX, United States; ^4^ Department of Oncology/Hematology, Houston Methodist Cancer Center, Houston, TX, United States

**Keywords:** cabozantinib, immunotherapy, pheochromocytoma, paraganglioma, nivolumab

## Abstract

**Introduction:**

Pheochromocytomas and sympathetic paragangliomas (PPGL) are neuroendocrine catecholamine-secreting tumors that are usually localized. Metastatic disease is rare and systemic treatment consists of conventional chemotherapy and high-specific-activity iodine-131-MIBG which was approved by the FDA in 2018. Although chemotherapy combinations still have value in specific settings, the debilitating side effects of treatment with only modest benefit have limited their use. With the introduction of a new generation of targeted therapy and immunotherapy patients with metastatic PPGL may have improved therapeutic options.

**Areas Covered:**

The current paper presents a case of a patient with metastatic PPGL who received multiple lines of systemic treatment. Despite progression on previous single agent cabozantinib and single agent pembrolizumab on separate clinical trials, the patient has exhibited a major response to the combination of cabozantinib and nivolumab for the past 22 months. In addition, we will review the available therapies for metastatic PPGL and discuss novel agents under clinical development.

**Conclusion:**

Newer targeted therapies and immunotherapy options are under clinical development with promising results for patients with PPGL.

## Introduction

Pheochromocytomas and sympathetic paragangliomas (PPGL) are catecholamine-secreting neuroendocrine tumors that arise from chromaffin cells of the adrenal medulla (in the case of pheochromocytomas) and the autonomic paraganglia (in the case of paragangliomas). The catecholamine excess produced by the majority of these tumors predisposes patients to cardiovascular and gastrointestinal morbidity and mortality (1, 2). Most PPGL are localized with 10% of the pheochromocytomas and more than 25% of the sympathetic paragangliomas undergoing metastatic spread of disease (3, 4). At present, there is no combination of clinical, histopathologic or biochemical features shown to reliably predict malignant behavior. As such, the diagnosis of a metastatic PPGL can only be made by identifying tumor deposits in tissues that normally do not contain chromaffin cells (e.g., lymph nodes, skeleton and brain) (5). Most frequent metastatic sites include the lymph nodes (80%), bone (71%), liver (50%), and the lungs (50%) (6). Among metastatic tumors the survival rate depends on the primary tumor site, the sites of metastases, the speed of progression, and the synthesis of catecholamines. Patients usually succumb from complications related to tumor burden (7).

At present, systemic treatment options include the consideration of conventional chemotherapy, radioactive iodine therapy for patients with uptake on the nuclear Iodine-131 meta-iodo-benzyl-guanidine (I^131^-MIBG) scan, and clinical studies. For patients with metastatic PPGL, the FDA approved high-specific activity Iodine-131 meta-iodo-benzyl-guanidine (I^131^-MIBG) in 2018 (8). In patients who present with *de novo* metastatic disease at initial presentation, a cytoreductive approach involving addressing the primary tumor is often pursued. The rationale behind this multidisciplinary approach is to de-bulk the extent of systemic tumor burden and decrease the catecholamine load. A retrospective review has suggested this approach may improve patient outcomes, but has not been prospectively validated (9).

Due to the rarity of this disease entity, prospective randomized trials in this rare disease are challenging and significant toxicity remains with the most commonly prescribed chemotherapy regimens (10–12). More recently, molecularly targeted and immunotherapeutic agents have been introduced with promising results (13–18). We report a patient with metastatic paraganglioma who received multiple targeted therapies and eventually had a dramatic, durable response to combination of cabozantinib and nivolumab. We also review the literature regarding current treatment options available for this rare disease. All the active clinical trials involving treatment options for metastatic PPGL are depicted in **Table 1**.

**Table 1:** Current clinical trials including agents used for patients with metastatic PPGL.

Agent	Eligible Patients	Phase	NCT#	Primary Outcome
I^131^-MIBG	Refractory neuroblastoma/metastatic PPGL	I	NCT03649438	Number of patients who receive MIBG
I^131^-MIBG	Refractory neuroblastoma or malignant PPGL	II	NCT00107289	Response rate
Lanreotide	Metastatic PPGL	II	NCT03946527	Tumor growth measurement
Sunitinib	Metastatic PPGL	II	NCT00843037	Clinical benefit rate^1^
Cabozantinib	Metastatic PPGL	II	NCT02302833	Response rate
Axitinib	Metastatic PPGL	II	NCT03839498	Response rate
Axitinib	Metastatic PPGL	II	NCT01967576	Response rate
Lenvatinib	Metastatic/advanced unresectable PPGL	II	NCT03008369	Response rate
ONC201	Neuroendocrine tumors	II	NCT03034200	Response rate
Temozolomide +/- Olaparib	Neuroendocrine tumor	II	NCT04394858	Progression Free Survival
Pembrolizumab	Rare unresectable or metastatic tumors	II	NCT02721732	Non-progression rate^2^

I^131^-MIBG, ^131^meta-iodo-benzyl-guanidine; PPGL, pheochromocytoma and paraganglioma. ^1^Clinical benefit rate is defined as either a partial response, complete response or stable disease for more than 12 weeks using Response Evaluation Criteria in Solid Tumors. ^2^Defined as the proportion of subjects in the analysis population who have no progression of disease at 27 weeks.

## Case Report

**Figure 1:**
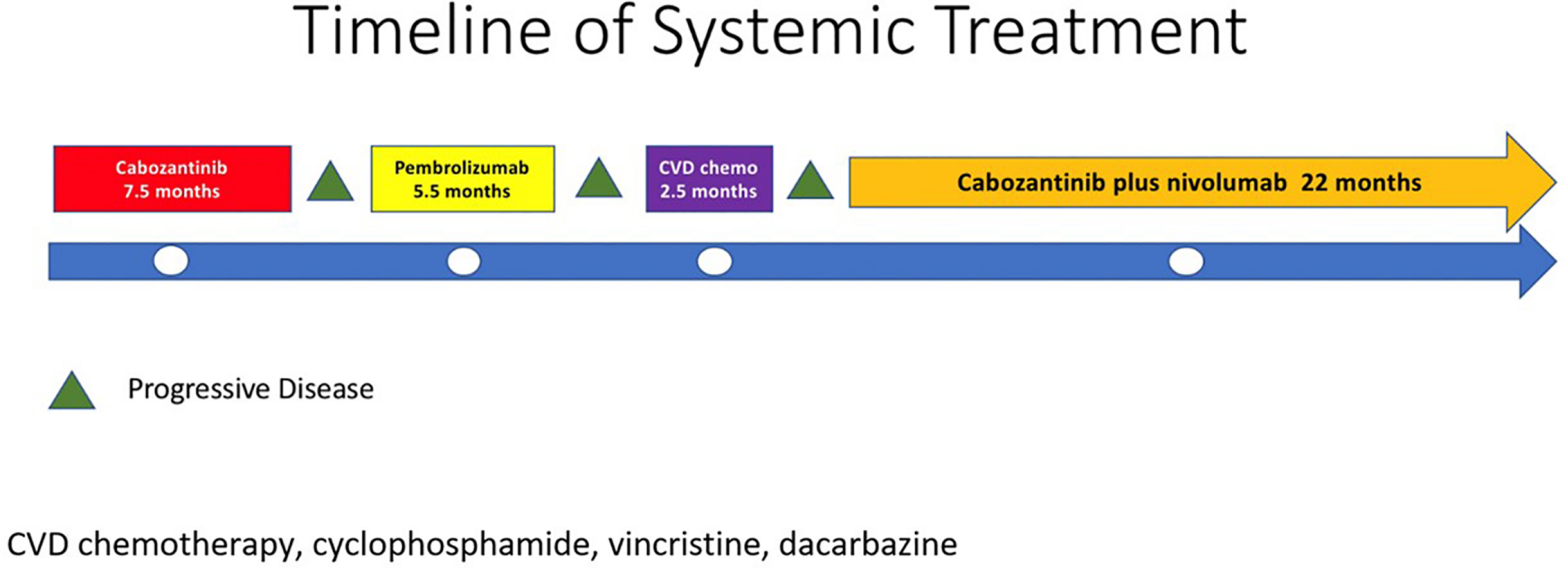
Timeline of systematic treatment.

**Figure 2:**
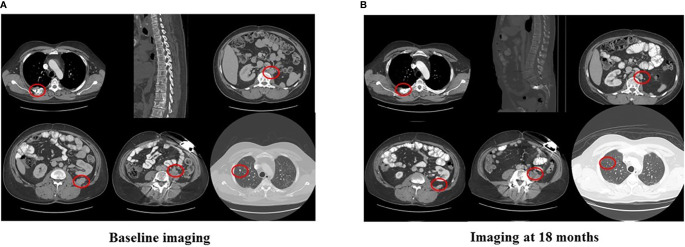
Imaging at baseline and after 18 months of nivolumab plus cabozantinib.

**Figure 3:**
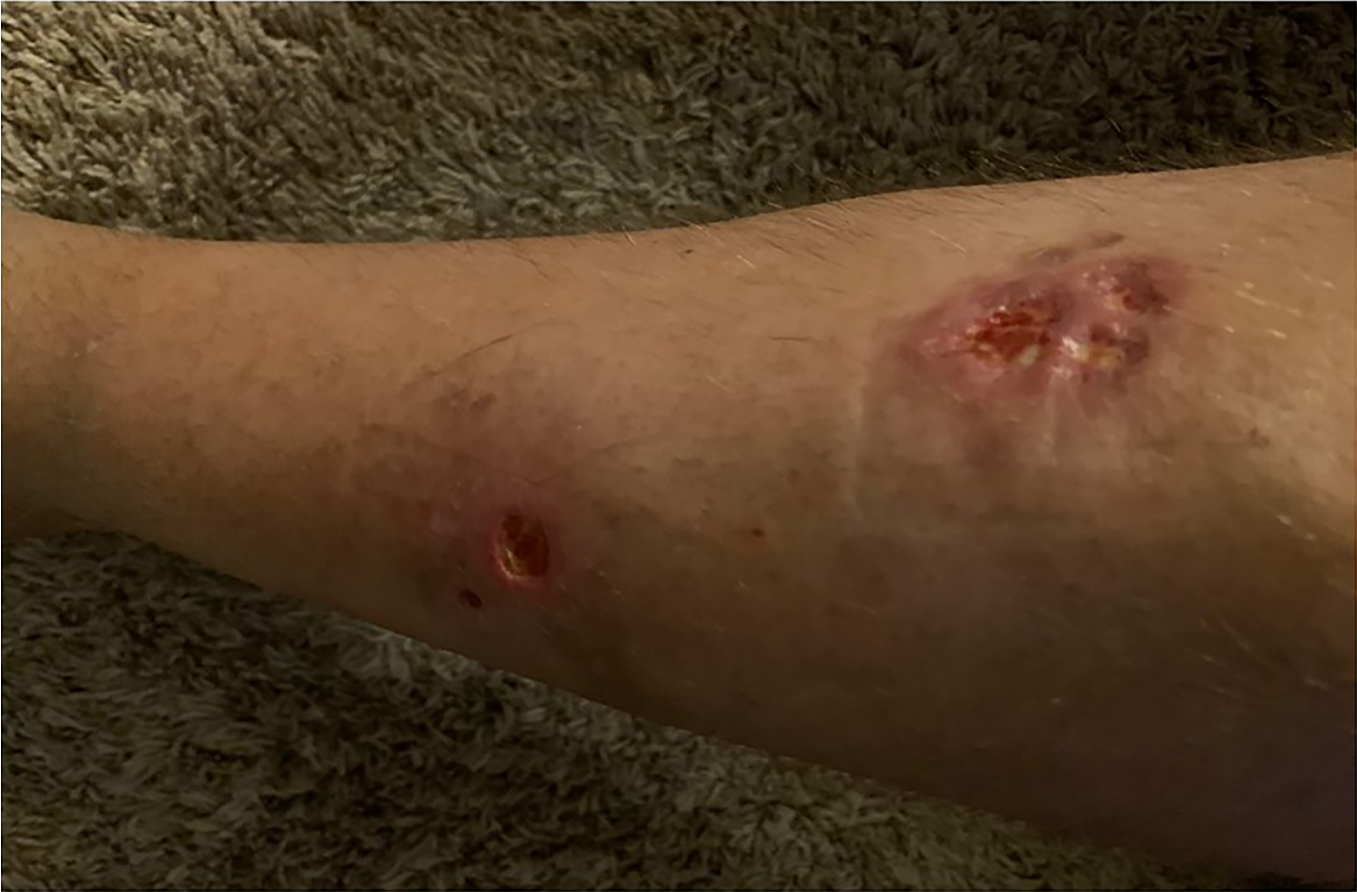
Right lower extremity shin ulcerations.

In 2007, a 32-year-old male patient was being worked up for a potential sinus surgery and was found to have a right sided neck mass. He underwent a surgical resection with histologic confirmation of a paraganglioma. Prior to his surgery he had no clinical evidence of catecholamine excess. In 2011, the patient underwent a scheduled surgery on his ankle which was complicated by a hypertensive crisis. He was found to have elevated plasma metanephrines and urinary catecholamines. On imaging work up, he was found to have a tumor involving his aorta originating from the organ of Zuckerkandl. After being started on phenoxybenzamine, he underwent surgery with resection of this tumor. In 2012, he was evaluated by a geneticist and reported no family history of malignancy. Genetic testing for *SDHB, SHDC*, and *SDHD* gene mutations by aCGH (ExonArrayDx) did not detect any disease-associated mutations in exons 1-8 of the *SDHB* gene, exons 1-6 of the *SDHC* gene, exons 1-4 of the *SDHD* gene, or the c.232 G>A in exon 3 of the *SDHAF2* gene (required for flavination of the SDHA subunit). He did not have detected mutations in the von Hippel-Lindau (*VHL*) *SDHA*, and Fumarase (*FH*) genes. In 2014 he suffered a transient ischemic attack with left-sided weakness that self-resolved within 24 h. At that time, his blood pressure was minimally elevated with systolic blood pressure in the 140–150 mm Hg range. In 2015, he was diagnosed with multiple metastatic lymph nodes involving his retroperitoneum encasing his left ureter. He again underwent surgical resection with eventual ureteral stent placement. In late 2015, he was found to have a metastatic tumor involving the caudate lobe of his liver for which he underwent trans-catheter arterial chemoembolization. In 2016, he underwent an octreotide nuclear scan of his abdomen. He was found to have a new metastatic lesion involving his sacrum and a left-sided skull-based lesion. In addition, a new retroperitoneal lesion was identified and the lesion involving the caudate lobe of the liver remained stable. The tumors showed I^131^-MIBG uptake. In 2016, he had resection of his caudate lobe and resection of the pre-aortic tumor. Soon after he was found to have disease progression in a para-aortic lymph node and within multiple bony lesions including his calvarium, thoracic spine, rib cage, and sacrum. He was enrolled in a clinical trial and received cabozantinib 60 mg by mouth daily on protocol. The patient was on study for 7.5 months prior to developing progressive disease in multiple known and new bone metastases, development of a new pulmonary metastasis, and progression of his known retroperitoneal lymph nodes. In 2017, the patient enrolled on a basket clinical trial with pembrolizumab and received 200 mg intravenously every 3 weeks for 5 months prior to experiencing disease progression in retroperitoneal and pelvic lymph nodes, new soft tissue metastasis within the abdomen, additional lung metastasis, and progression of multiple bone metastasis including the development of a new femoral lesion. His best response on the study was considered stable disease. He then received three cycles of cyclophosphamide, vincristine, and dacarbazine. His treatment course was complicated by severe myelosuppression. He required admission for neutropenic fever and received multiple transfusions of both packed red blood cells and platelets. With chemotherapy he had evidence of disease response with a 12.2% reduction in his measurable disease with stability in his bone metastasis. However, within 3 months of ending chemotherapy he had progressive pain in his back and was found to have progression of disease in vertebral column bone metastases. A timeline of his systemic treatment is provided in **Figure 1**. The patient was started on off label cabozantinib and nivolumab. Cabozantinib was dosed at 40 mg by mouth daily and nivolumab was provided at 240 mg IV every 2 weeks as per the phase I study by Apolo et al. (19) **Figure 2** contrasts his baseline imaging studies with the ones obtained 18 months after treatment initiation. The patient’s course was complicated by several events including ulcerations on his lower extremities shown in **Figure 3**. A biopsy of these ulcerations found evidence of mild epithelial spongiosis with focal parakeratosis. There was evidence of a superficial perivascular lymphocytic infiltrate with scattered eosinophils. The dermatopathology group at MD Anderson Cancer Center determined these findings were most consistent with a hypersensitivity reaction to an internal medication. Breaks from cabozantinib allowed these ulcerations to heal. At present the patient remains on this regimen 22 months after initiation with evidence of continued clinical benefit. He had significant tumor reduction with decrease in size of lung, abdominal, bone and retroperitoneal lesions. On treatment his plasma metanephrines decreased from a baseline level of 14 nmol/L to 3.6 nmol/L. In addition, the patient symptomatically improved without signs of catecholamine excess and his blood pressure remains very well controlled after being started on both alpha and beta blockers.

## Discussion

When considering this gentleman’s case, several interesting observations can be rapidly gleaned. First, the patient had been heavily pre-treated prior to receiving the combination of cabozantinib and nivolumab. The second observation was the greatly enhanced activity of the combination of cabozantinib and nivolumab as compared to when he received either single agent cabozantinib or pembrolizumab on two protocols. The third and final observation are the potential challenges in toxicity that can emerge with new treatments alone or in combination. In the following discussion we will review the currently available systemic treatment options and will provide additional information in regards to both targeted therapeutics and immunotherapy that have prospective data and highlight ongoing studies.

### Cytotoxic Chemotherapy

Systemic chemotherapy should be considered for patients with unresectable and rapidly progressive metastatic/unresectable PPGL and in patients with high tumor burden. The most extensively used chemotherapeutic agents include cyclophosphamide, vincristine, dacarbazine (CVD) (12, 20–22). In a retrospective study evaluating 54 patients treated with chemotherapy at MD Anderson Cancer Center, 33% of patients had a decrease in tumor size and achieved blood pressure control (12). In addition, a meta-analysis performed by Niemeijer et al. reported an objective response rate of 37% and a partial response in catecholamine excess in 40% of patients receiving CVD (10).

### Radionuclide Therapy

Radionuclide therapy has been long used for treatment of metastatic PPGL and consists mainly of I^131^-MIBG. I^131^-MIBG was first introduced in the 1980s as a potential therapy for PPGL that express the norepinephrine transporter (NET) in their cell membranes (23). Like norepinephrine, I^131^-MIBG is captured by the NET. With increasing dose levels, I^131^-MIBG emits sufficient radiation to lead to cellular damage. In an effort to improve the activity and the toxicity profile of I^131^-MIBG, high-specific-activity (HSA I^131^-MIBG) was introduced and the recommended phase II dose was determined (24). In the pivotal phase II study of HSA I^131^-MIBG, the primary endpoint was the reduction in the number of anti-hypertensives and anti-hypertensive dose by greater than or equal to 50%. The primary endpoint was achieved in 25% of the population. An impressive 92% of patients had disease control as best response as defined by the proportion of patients with complete response, partial response, and stable disease as per RECIST v1.1 criteria (25). While myelosuppression was again witnessed, no patients required autologous stem cell rescue and no patients experienced a hypertensive crisis during the delivery of therapy. These findings led the FDA to approve HSA I^131^-MIBG for patients with metastatic PPGL in 2018 and has become the de facto standard of care.

### Molecularly Targeted Therapy

Small molecule TKIs targeting the vascular endothelial growth factor receptor (VEGFR) are well established in the treatment of metastatic renal cell carcinoma including sorafenib, sunitinib, pazopanib, axitinib, cabozantinib, and lenvatinib (26). In addition to VEGFR, significant activity against other important targets in malignancy including the fibroblast growth factor receptors for lenvatinib and c-Met for cabozantinib (27). Early reports suggest the utility of these agents in metastatic PPGL (13–15, 28, 29). The largest retrospective series included 17 patients with metastatic PPGL who were treated with sunitinib monotherapy (15). Of 14 evaluable patients, three (21%) had a partial response and five (36%) had stable disease. The median progression-free survival was 4.1 months. Six patients (43%) had a reduction in catecholamines. Finally, in a recent phase II trial of sunitinib in patients with PPGL, 25 patients with progressive disease were enrolled and received sunitinib at a dose of 50 mg daily 4 weeks on 2 weeks off. The median progression free survival was 13.4 months and three patients (13%) with germline mutations (SDHB) achieved partial remissions (18).

The most worrisome adverse effect of TKIs in patients with PPGL is exacerbation of pre-existing secondary hypertension. Patients with PPGL already have elevated blood pressure due to catecholamine excess. A class effect of TKIs targeting VEGFR is hypertension. Suggested management of hypertension in patients with PPGL can be found in a review by Jasim and Jimenez (30).

Sunitinib is currently being studied in an open label phase II study for patients with metastatic PPGL (NCT00843037). Axitinib is being studied in a two phase II trials in the same population (NCT03839498, NCT01967576). Cabozantinib is used as monotherapy in an active phase II clinical trial (NCT02302833). In addition, lenvatinib is being evaluated in a phase II study in patients with metastatic or advanced unresectable PPGL (NCT03008369).

### Immunotherapy

The discovery of immune checkpoints and the development of antibodies targeting cytotoxic lymphocyte antigen 4 (CTLA-4), programmed death receptor 1 (PD-1), and programmed death receptor ligand 1 (PD-L1) has resulted in a paradigm shift in the treatment of both solid and liquid malignancies. In a recent phase II clinical trial of pembrolizumab (anti-PD-1) in advanced rare cancers, 11 patients with metastatic PPGL were evaluated (31). Of the 11 patients, four patients had germline mutations and seven of the patients were hormonally active. The progression free survival (PFS) at 27 weeks after initiation of therapy was 40%, the median PFS was 5.7 months. The overall response rate was 9%.

### Combination of TKI and Immune Checkpoint Therapy in Other Malignancies

In metastatic renal cell carcinoma (mRCC), the combination of axitinib (targeting VEGFR 1,2,3) with both pembrolizumab (KEYNOTE 426 study) and avelumab (JAVELIN 101 study) have both received FDA approval based on pivotal phase III trials (32, 33). The combination of cabozantinib and nivolumab (anti-PD-1) with or without ipilimumab (anti-CTLA-4) has also been explored in patients with genitourinary malignancies (19). The combination was found to have activity across a variety of tumors including multiple rare genitourinary tumors including rare variants of urothelial cancer and penile cancer. Recently, the initial results of a phase III randomized study of cabozantinib plus nivolumab versus sunitinib as initial therapy for mRCC (NCT03141177) found a significant improvement in both progress survival and overall survival (Choueiri T et al). The combination of lenvatinib and pembrolizumab recently received FDA for the treatment of advanced endometrial cancer after a phase IB/II study found a 38% overall response rate in previously treated patients (34). This response rate was favorable as compared to a single agent study of lenvatinib which found an overall response rate of 14% (35) and of single agent pembrolizumab finding a response rate of 13% (36).

Significant debate has emerged if TKI plus immune checkpoint therapy leads to additive or synergistic impact. Besides the extrapolation from other malignancies, the use of TKIs and immunotherapy might have a plausible mechanistic rationale in the treatment of PPGL. In surgically resected PPGL up to 50% of tumors express PD-L1/PD-L2 (37). TKIs inhibit multiple endothelial growth factors that prevent neo-angiogenesis. Cabozantinib is a potent antiangiogenic medication used in clinical practice targeting VEGFR2, c-MET, Axl, and Ron (36). Targeting c-Met may be particularly relevant to metastatic PPGL since activating mutations of the MET gene have recently been described (38, 39). Furthermore, cabozantinib may induce vascular normalization that facilitates the recognition of the tumor cells by the immune system (Reference: Jimenez C, Antiangiogenic therapies for pheochromocytoma and paragangliomas, Endocrine Related Cancer, 2020).

In our patient, it is impossible to know if he would have this tremendous durable response if he had received the combination initially or if chemotherapy prior to treatment potentially enhanced the benefit. However, in multiple other tumor types including renal cell carcinoma and urothelial carcinoma, response rates have been numerically less when immunotherapy is provided either post TKI (40) vs as upfront therapy (41) or post chemotherapy in urothelial cancer (42, 43). Given the activity seen in metastatic PPGL with TKI therapy and with immune checkpoint therapy in small studies, we feel a prospective combination study of cabozantinib plus immune checkpoint therapy is warranted.

## Conclusion

The development of tyrosine kinase inhibitors and immunotherapy in common cancers has led to exploration of activity in patients with metastatic PPGL. Despite the significant improvement in our understanding of the disease and the exciting approval of HSA I^131^ MIBG, patients with metastatic PPGL continue to present multiple therapeutic challenges. The clinical heterogeneity of metastatic PPGL patients necessitates a deep understanding of the molecular features of the disease in order to individualize treatment and improve outcomes. While the backbone of chemotherapy will still be used in patients with rapidly progressive disease there is substantial hope that targeted therapy, immunotherapy, and combination approaches will improve the outcomes for patients with this rare disease.

## Data Availability Statement

The raw data supporting the conclusions of this article will be made available by the authors, without undue reservation.

## Ethics Statement

Written informed consent was obtained from the individual(s) for the publication of any potentially identifiable images or data included in this article.

## Author Contributions

All authors contributed to the drafting of this manuscript and agree with the details included. All authors contributed to the article and approved the submitted version.

## Conflict of Interest

AS: Research grant support from Exelixis, Bristol Myers Squibb, Merck. CJ, MH, MC: Research grant from Exelixis.

The remaining authors declare that the research was conducted in the absence of any commercial or financial relationships that could be construed as a potential conflict of interest.

The reviewer LC declared a past co-authorship with one of the authors CJ to the handling editor.
